# The Endothelial Mechanotransduction Protein Platelet Endothelial Cell Adhesion Molecule-1 Is Influenced by Aging and Exercise Training in Human Skeletal Muscle

**DOI:** 10.3389/fphys.2018.01807

**Published:** 2018-12-18

**Authors:** Lasse Gliemann, Nicolai Rytter, Peter Piil, Jannik Nilton, Thomas Lind, Michael Nyberg, Matthew Cocks, Ylva Hellsten

**Affiliations:** ^1^Department of Nutrition, Exercise and Sports, Section for Integrative Physiology, University of Copenhagen, Copenhagen, Denmark; ^2^Exercise Metabolism Research Group, School of Sport and Exercise Sciences, University of Birmingham, Birmingham, United Kingdom

**Keywords:** vascular function, passive leg movement, mechanosensors, aging – old age – seniors, shear stress

## Abstract

**Aim:** The aim was to determine the role of aging and exercise training on endothelial mechanosensor proteins and the hyperemic response to shear stress by passive leg movement.

**Methods:** We examined the expression of mechanosensor proteins and vascular function in young (*n* = 14, 25 ± 3 years) and old (*n* = 14, 72 ± 5 years) healthy male subjects with eight weeks of aerobic exercise training. Before and after training, the hyperaemic response to passive leg movement was determined and a thigh muscle biopsy was obtained before and after passive leg movement to assess the acute effect of increased shear stress. Biopsies were analyzed for protein amount and phosphorylation of mechanosensor proteins; Platelet endothelial cell adhesion molecule-1 (PECAM-1), Vascular endothelial cadherin, Vascular endothelial growth factor receptor-2 and endothelial nitric oxide synthase (eNOS).

**Results:** Before training, the old group presented a lower hyperaemic response to passive leg movement and a 35% lower (*P* < 0.05) relative basal phosphorylation level of PECAM-1 whereas there was no difference for the other mechanosensor proteins. After training, the eNOS protein amount, the amount of PECAM-1 protein and the passive leg movement-induced phosphorylation of PECAM-1 were higher in both groups. The hyperaemic response to passive leg movement was higher after training in the young group only.

**Conclusion:** Aged individuals have a lower hyperaemic response to passive leg movement and a lower relative basal phosphorylation of PECAM-1 than young. The higher PECAM-1 phosphorylation despite a similar hyperemic level in the aged observed after training, suggests that training improved shear stress responsiveness of this mechanotransduction protein.

## Introduction

Impaired vascular function is a hallmark of aging (Seals et al., 2011) and vascular dysfunction is a strong predictor of cardiovascular disease (Widlansky et al., 2003; Deanfield et al., 2007; Green et al., 2011). The decline in vascular function associated with aging has been proposed to be related to a dysfunctional NO system (Taddei et al., 2006). However, protein levels of eNOS are not lower in old subjects compared to younger subjects (Donato et al., 2009; Nyberg et al., 2012b) indicating that the impairment in the NO system is related to activation of eNOS and/or subsequent inactivation of NO (Gryglewski et al., 1986; Guzik et al., 2002; Fleming and Busse, 2003). Shear stress, the frictional force exerted on the endothelial layer by the flowing blood, is known to be important for vascular function and cell culture studies have identified a mechanosensor protein complex which activates eNOS during experimental shear stress stimulation of endothelial cells. In cultured endothelial cells, shear stress induces phosphorylation of vascular VEGF-R2, vascular endothelial cadherin (VE-cadherin) and PECAM-1 (Osawa et al., 2002; Shay-Salit et al., 2002; Tzima et al., 2005) which in turn causes phosphorylation and activation of eNOS (Fleming and Busse, 1999; Jin et al., 2003; Fleming et al., 2005; Tzima et al., 2005). Of these mechanosensor proteins, PECAM-1 has consistently been shown to be very important for endothelial cell function (Privratsky and Newman, 2014) but evidence for how mechanosensor proteins are influenced in humans is lacking. *In vivo* in humans, the vascular response to increased flow and thereby shear stress can be evaluated by examining the hyperaemic response to passive leg movement (Hellsten et al., 2008; Mortensen et al., 2012). Passive leg movement causes an NO dependent increase in blood flow to the leg without an influence of an increase in muscle activity or metabolism (Hellsten et al., 2008; Mortensen et al., 2012). Studies on young subjects and older adults have shown that the hyperaemic response to passive leg movement is dependent on NO in both young and older subjects but that the hyperaemic response is reduced with aging (Mortensen et al., 2012; Trinity et al., 2015).

We have recently found that vascular function, assessed *in vivo* by passive leg movement, and alterations in the mechanosensor complex, can be impaired in young healthy subjects by two weeks of high sugar intake (Gliemann et al., 2017). Based on these observations and the substantial evidence for the role of endothelial mechanosensors from *in vitro* models (Osawa et al., 2002; Shay-Salit et al., 2002; Tzima et al., 2005), we hypothesized that alterations to the mechanosensor complex is a potential mechanism contributing to the impaired vascular function in older subjects.

Exercise training is known to be beneficial for vascular function and indications from cross-sectional studies suggest that regular exercise training can counteract the age-related decline in vascular function (DeSouza et al., 2000; Taddei et al., 2000; Nyberg et al., 2012a). The positive effects of exercise training on vascular function have been attributed to the NO system (DeSouza et al., 2000; Taddei et al., 2000; Nyberg et al., 2012a) but there is a paucity of knowledge on how activation of the NO system is influenced by training. Here we evaluated the influence of training on the expression and activation of the mechanosensor complex and of eNOS in young and old men, to assess whether mechanically induced activation of eNOS can be improved by training.

The hypotheses of the present study were that (a) the lower hyperemic response to shear stress in older men is associated with impairments in the endothelial mechanosensor complex and eNOS activation and (b) shear stress induced hyperemia is improved after a period of exercise training and paralleled by improved ability of the mechanosensor proteins to sense and respond to shear stress resulting in increased eNOS phosphorylation.

## Materials and Methods

### Ethics Statement

The study was approved by the Ethics Committee of Copenhagen and Frederiksberg communities (H-6-2014-085) and was conducted in accordance with the latest guidelines of the Declaration of Helsinki. Written informed consent was obtained from all subjects before enrolment in the study.

### Subjects

Fourteen young (20–28 years) and 14 older (66–80 years) healthy men participated in the study. Before enrolment in the study, all subjects were screened by electrocardiography and their medical history and level of daily physical activity were registered. All included subjects were non-smokers without any known diseases, and were not engaged in regular intense physical activity or training (<2 weekly sessions of moderate to intense exercise).

### Exercise Training Intervention

Each subject completed 8 weeks combined training consisting of two weekly intense bicycle ergometer (spinning) sessions and 1 cross-training session (combined functional strength training and intermittent intense aerobic training). Briefly, spinning sessions consisted of repeated, intense (>80% maximal heart rate) 5-min intervals separated by 1–2-min active recovery periods. Training intensity and duration was progressively increased with three intervals during training week 1 and 2 and 5 intervals during week 3–8. Training intensity was monitored with an online heart rate monitoring system during each session (Polar Team2, Polar Electro Oy, Kempele, Finland). The heart rate response was subsequently analyzed (Polar ProTrainer 5 software) for determination of heart rate response and distribution during training. Both groups showed similar training compliance during the 8 week intervention, and training intensity was equally high in both groups (Table 2). One subject was excluded due to low training compliance (<60%).

### Pre-testing

Before the first experimental day subjects visited the lab for determination of body composition and peak pulmonary oxygen uptake (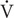
O_2peak_) and for familiarization with the passive leg movement on an one-leg knee-extensor model. Body composition was determined in the morning in a fasted state with whole-body dual-energy X-ray absorptiometry scanning (DEXA; Prodigy, GE Healthcare, Brøndby, Denmark). The passive leg movement was performed on a knee extensor ergometer (Andersen and Saltin, 1985).

Pulmonary 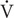
O_2peak_ was determined (Oxycon Pro, Viasys Healthcare, Höechberg, Germany) with an incremental exercise test on a mechanically braked cycle ergometer (Monark Ergomedic 839E, Monark, Sweden). The incremental exercise test consisted of a 4-min submaximal bout at 50 and 100 W for old and young, respectively. Subjects then rested for 2 min after which the incremental test began at the work load corresponding to the submaximal bout and increased by 25 W min^−1^ until volitional exhaustion. 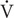
O_2peak_ was calculated as the average of the three highest consecutive 15 s values. Recognition of true 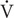
O_2peak_ required three of the following five criteria to be met: Individual perception of exhaustion, respiratory exchange rate (RER) >1.15, VO_2_ curve plateau, heart rate approaching age-predicted maximum, or inability to maintain pedaling frequency above 80 RPM. Verbal encouragement was offered throughout the test. Heart rate was measured throughout the exercise protocol (Polar Team2, Polar Electro Oy, Kempele, Finland).

### Experimental Days

Subjects arrived at the laboratory in the morning after an overnight fast. While subjects rested in the supine position, a catheter (DB Venflon Pro Safety, 18 GA, Becton Dickinson Infusion Therapy AB, Sweden) was inserted into the antecubital vein for blood sampling. Blood was drawn at rest and after 20 min of passive leg movement (Mortensen et al., 2012). Two microdialysis probes (CMA63 with 30 mm membrane length and 20 kDa cutoff, M Dialysis AB, Sweden) were inserted into the m. vastus lateralis under local anesthesia (Xylocain, ∼3 ml, 20 mg ml^−1^, AstraZeneca, Sweden). After insertion of the probes, subjects rested for 120 min to allow the muscle to attain a resting state and to recover from insertion trauma. The microdialysis probes were perfused with Ringer acetate buffer (Fresenius Kabi AB, Norway) at a rate of 5 μl min^−1^ and to determine the relative exchange over the membrane, a small amount (2.7 nM) of [2–3H] labeled adenosine was added to the perfusate to allow for calculation of probe recovery. Microdialysate was collected during the final 20 min of the 120 min rest, excluding the first 2 min to account for delay in the probe perfusate. Immediately after collection, samples were weighed and triplicates of 5 μl of dialysate were allocated into 3 ml Ultimate Gold scintillation liquid (PerkinElmer, Waltham, MA, United States). The [2-^3^H] adenosine activity of the dialysate was measured on a liquid scintillation counter (Tri-Carb 2910 TR, PerkinElmer, Waltham, MA, United States). The remaining dialysate was immediately frozen at –80°C. Probe recovery (PR) was calculated as [PR = (dpm_infusate_ – dpm_dialysate_/dpm_infusate_)], where dpm denotes disintegrations per minute (Scheller and Kolb, 1991; Jansson et al., 1994). The probe recovery was used to obtain an estimate of probe recovery and the resulting values are accordingly presented as estimated interstitial concentrations.

Passive leg movement was conducted by the subject being seated in a knee extensor model and the lower leg being moved back and forth in a kicking movement by one of the investigators at a frequency of 60 cycles per minute (Hellsten et al., 2008). The subject was instructed to completely relax the muscles of the leg during the session. Femoral arterial blood flow was measured at rest and during the first 5 min of passive leg movement by use of ultrasound Doppler (GE Vivid E9, GE Healthcare, Pittsburgh, PA, United States) equipped with a linear probe (L9) operating at an imaging frequency of 4/8 MHz and a Doppler frequency of 4.2 MHz. The site of blood velocity measurements in the common femoral artery was distal to the inguinal ligament but above the bifurcation into the superficial and profound femoral branch to avoid turbulence from the bifurcation. All recordings were obtained at the lowest possible insonation angle and always below 60°. The sample volume was maximized by choosing the widest section of the vessel and the measurements were made without interference of the vessel walls. A low-velocity filter (velocities < 1.8 m s^−1^) rejected noise caused by turbulence at the vascular wall. Doppler tracings and B-mode images were recorded continuously and Doppler tracings were averaged over 45 s. Resting femoral arterial blood flow was measured before passive leg movement. During the 5 min passive leg movement, femoral arterial blood flow was measured at 15 s intervals during the initial 3 min, and after 4 and 5 min. Peak blood flows and mean blood flow for the first 5 min are reported herin.

Before and immediately the passive leg movement bout, a muscle biopsy was obtained from the m. vastus lateralis under local anesthesia (Xylocain, ∼5 ml, 20 mg ml^−1^, AstraZeneca, Sweden) using the percutaneous needle biopsy technique with suction (Bergström, 1975). The biopsies were immediately frozen in liquid nitrogen or embedded in Tissue-Tek OCT compound (Sakura Finetek, Zoeterwoude, Netherlands) and frozen in liquid nitrogen cooled isopentane. Frozen samples were stored at 80°C until further analysis.

All tests and experimental days were performed before and after the 8-week training intervention.

### Quantification of Protein Expression

Biopsies were freeze-dried and dissected free from fat, blood and connective tissue. Approximately 5 mg dry weight of muscle tissue were homogenized in a fresh batch buffer (10% glycerol, 20 mM sodium-pyrophosphate, 150 mM NaCl, 50 mM HEPES, 1% Nonidet P-40, 20 mM β-glycerophosphate and proteolytic inhibitors) two times for 30 s (Qiagen Tissuelyser II, Retsch, Haan, Germany). After rotation end-over-end for 1 h, the samples were centrifuged for 30 min at 17,500 × *g* at 4°C and the lysates were collected as the supernatant. Protein concentrations were determined in the lysates using BSA standards (Pierce Reagents, Rockford, IL, United States). The lysates were diluted to appropriate protein concentrations in a concentrated sample buffer (0.5 M Tris-base, DTT, SDS, glycerol, and bromphenol blue), and equal amount of total protein were loaded for each sample in different wells on precasted Tris–HCL gels (Bio-Rad, Hercules, CA, United States). For comparisons, samples from the same subject were always loaded on the same gel. After gel electrophoresis, the proteins were transferred (semidry) to a polyvinylidene difluoride membrane (Immobilon Transfer Membrane, Millipore), which was incubated with ∼10 ml of primary antibody over-night and then washed three times for 5 min in Tris-buffered saline-Tween before incubation with secondary antibody for 1 h. The membranes were incubated with the following primary antibodies: VEGF-R2 (sc-19530; Santa Cruz Biotechnology, Santa Cruz, CA, United States); VEGF-R2^Tyr1175^ (2478, Cell Signaling Technology, Danvers, MA, United States); eNOS (BD 610287, BD Biosciences, San Jose, CA, United States; eNOS^ser1177^ (Calbiochem 482737, Merck Milipore, Darmstadt, Germany); PECAM-1 (AF806, R&D Systems Inc., Minneapolis, MN, United States); PECAM-1^Tyr713^ (BS4666, Bioworld Technology, St. Louis Park, MN, United States); VE-cadherin (ab33168, Abcam, Cambridge, United Kingdom); VE-cadherin^Tyr731^ (NBP1-51393, Novus Biologicals, Littleton, CO, United States). Secondary antibodies used were goat anti-rabbit or rabbit anti-goat HRP-conjugated antibodies (P-0448 and P-0449; DakoCytomation, Glostrup, Denmark; 1:5000). Following detection and quantification (ChemiDoc MP system, Bio-Rad, Hercules, CA, United States), the protein content was expressed in arbitrary units. The blots were also analyzed for glyceraldehyde 3-phosphate dehydrogenase (GAPDH; ab9484; Abcam), actin (A2066; Sigma-Aldrich, St. Louis, MO, United States) or alpha tubulin (ab4076, Abcam) depending on molecular weight of the protein that was studied to check for loading inconsistencies (Figure 3E). Due to numerous biological factors influencing GAPDH and alpha tubulin (Lowe et al., 2000; Galpin et al., 2012) the specific proteins analyzed have not been normalized to the housekeeping genes. The total protein amount of the samples has been determined by Stain-Free imaging and quantification (Vigelsø et al., 2015) and representative Stain-Free images are included in the Supplementary Material. In the case of loading inconsistency, the western blood procedure were repeated. Quantification of the total protein amount across all samples of the study show that there is no significant difference in protein amount between the young and the old group. Reported values are unadjusted densitometry values.

### Quantitative Immunofluorescence Analysis of eNOS and eNOS^ser1177^ Phosphorylation

The procedures for immunofluorescence staining and subsequent image analysis has been adapted from the original method described by Cocks et al. (Cocks et al., 2012). Transverse orientated serial 5 μm sections were cut using a microtome. Four sections from each condition were collected on to room temperature uncoated glass slides. Sections were fixed in acetone and ethanol (3:1) and then dual stained with antibodies against eNOS (BD 610297, BD Biosciences, San Jose, CA, United States) and eNOS^ser1177^ (Cell Signaling Technology 957, Beverly, MA, United States). Sections were then incubated with goat anti-mouse IgG1 546 (eNOS) and goat anti-rabbit IgG 633 (eNOS^ser1177^) secondary antibodies (A-21123 and A-21070; ThermoFisher Scientific, San Jose, CA, United States), in combination with Ulex europaeus I lectin–FITC conjugate (UEA-I–FITC; Sigma-Aldrich L9006, St. Louis, MO, United States) as a marker of the endothelium. Coverslips were then applied using a glycerol and Mowiol 4-88 solution.

Images were acquired using an inverted confocal microscope (Zeiss LSM-710, Carl Zeiss, Germany) with a 40× oil immersion objective. FITC fluorescence was excited with a 488 nm multiline argon laser and detected with 493–557 nm emission. Alexa Fluor 546 and 633 fluorophores were excited with 543 and 633 nm lines of the helium–neon laser and 548–623 and 638–747 nm emission, respectively. The images were acquired at a resolution of 1,024 × 1,024 pixels and stored in 24-bit tagged image format file format. Identical settings were used for all image capture within each participant. The mean number of views assessed was 6 ± 2. The number of capillaries quantified was approx. 30.

Image analysis was performed using Image Pro Plus 5.1 software. Only within participant differences were assessed. Endothelial specific fluorescence was determined using the UEA-I–FITC endothelial marker image, which was extracted and overlaid onto the corresponding eNOS and eNOS^ser1177^ images. Fluorescence intensity was then quantified within the endothelial specific area. The mean fluorescence intensity of Pre-training Pre-passive movement samples were normalized to a value of 1, and the relative intensity of subsequent samples was calculated. In addition, as eNOS and eNOS^ser1177^ were dual stained it allowed for assessment of eNOS/eNOS^ser1177^ ratio on an individual vessel basis to be calculated. Changes in eNOS/eNOS^ser1177^ ratio from the immunohistochemical analysis were used to detect changes in phosphorylation status going from rest to passive leg movement. The immunofluorescence analysis employed allowed for assessment of eNOS phosphorylation specifically within the endothelium, and as such a more sensitive assessment of changes to the passive leg movement than would be allowed using western blotting on whole muscle homogenates (Cocks et al., 2012). We have however, also conducted western blot analysis of eNOS and eNOS phosphorylation. These data are in the Supplementary Material.

**Table 1:** Subject characteristics before and after 8 weeks aerobic high-intensity exercise training.

	Young (*n* = 14)			Old (*n* = 13)		
Variable	Before	After	Δ%	Before	After	Δ%
Age (years)	25 ± 3			72 ± 5
Weight (kg)	80.7 ± 13.9	80.0 ± 14.2	0.9 ± 3.4	79.8 ± 9.2	78.5 ± 10.7^∗^	1.8 ± 2.3
Body fat (%)	25.1 ± 7.4	23.3 ± 7.3^∗^	6.9 ± 10.7	27.6 ± 5.1	26.3 ± 4.0^∗^	4.0 ± 5.6
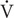 O_2peak_ (ml min^−1^)	3559 ± 497	3805 ± 460^∗^	7.4 ± 8.2	2414 ± 293#	2527 ± 404#^∗^	4.4 ± 7.3
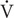 O_2peak_ kg^−1^ (ml min^−1^kg^−1^)	44.8 ± 7.2	48.3 ± 6.9^∗^	8.4 ± 6.9	30.4 ± 3.0#	32.4 ± 4.1#^∗^	6.3 ± 7.1

*Data are presented as mean ± SD. 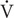
O_2peak_, maximal oxygen uptake; HR, heart rate. #Significantly different from the young group (P < 0.05); ^∗^Significantly different from before training (P < 0.05).*

### Analysis of NOx in Plasma and Microdialysate

The stable metabolites of NO, nitrite and nitrate (NOx) in plasma and microdialysate, were measured using a fluorometric EIA kit (Cayman Chemical Co., Ann Harbor, MI, United States). Interstitial concentrations were estimated based on the relative recovery of [2-^3^H] adenosine in the microdialysis probe. Based on previous findings it was assumed that the relative recovery of [2-^3^H] adenosine is similar to the recovery of other compounds (Höffner et al., 2003).

### Statistical Analysis

Subject number is based on power calculations of selected primary outcome measures. These include average differences and variability in femoral arterial blood flow in response to passive leg movement and protein expression and phosphorylation of mechanosensors and eNOS in muscle homogenates from m. vastus lateralis (Altman, 1980). Significance level for all tests was set at an *a*-level of *P* < 0.05 at a power level of 0.8. Data are means ± SD.

A linear mixed-model approach (R-studio, Version 0.99.903, R Foundation for Statistical Computing, Vienna, Austria) was used to investigate differences within and between groups. Fixed factors were “group” (young or old), “intervention” (before or after training) and “time” (rest or passive leg movement). Subjects were specified as a repeated factor and identifier of random variation. The homogeneity of variance and normal distribution was confirmed through residual and Q–Q plots. Pairwise differences were identified using the unadjusted difference *post hoc* procedure.

## Results

### Subjects Characteristics and Training Intervention

Before the training period, 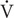
O_2peak_ and 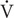
O_2peak_ kg^−1^, were higher (*P* < 0.05) in the young compared to the old group (Table 1). Exercise training increased (*P* < 0.05) both variables in the young and the old group. Both groups showed similar compliance with the training intervention (Table 2). These data have previously been published (Piil et al., 2018).

### Blood Flow Response to Passive Leg Movement

**FIGURE 1:**
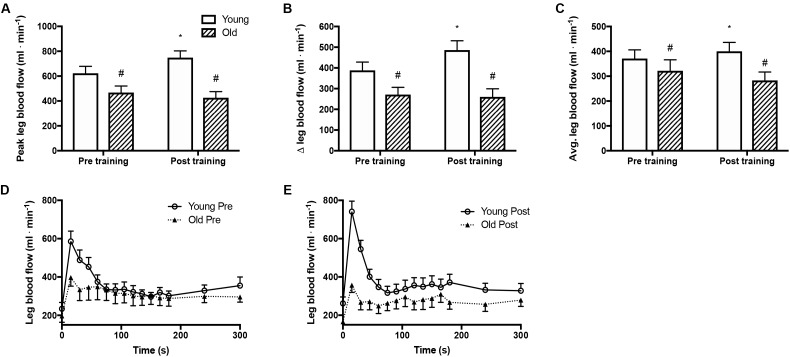
Femoral arterial blood flow during passive leg movement. **(A)** Peak femoral arterial blood flow in response to passive leg movement, **(B)** changes from rest to passive leg movement in femoral arterial blood flow, **(C)** average femoral arterial blood flow during passive leg movement and **(D,E)** time curse of femoral arterial blood flow in young and old subjects before and after the training intervention. #denotes difference from young group, ^∗^denotes difference from before training within group. Data are mean ± SD.

Peak and mean femoral arterial blood flow in response to passive movement was 25% lower (*P* = 0.005, Figures 1A,C) in the old than the young group before training and was increased with training by 20% (*P* = 0.006) in the young group but not in the old. The peak change in blood flow from rest to passive movement was also lower (*P* < 0.001, Figure 1B) in the old than in the young group before training and was increased after the training period in the young group only (*P* = 0.039). Blood flow traces from both groups before and after training are presented in Figures 1D,E. While blood flow are reported as absolute values, normalizing blood flow to leg mass does not significantly change the outcome.

### Comparison of Basal Expression and Phosphorylation of eNOS and Mechanosensor Protein

#### eNOS

**FIGURE 2:**
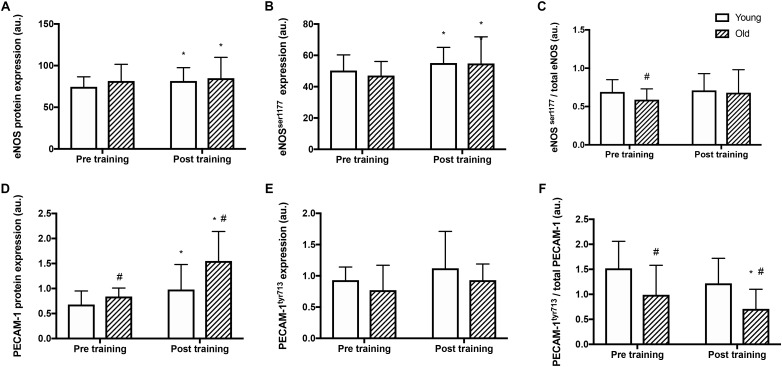
Protein expression and relative phosphorylation of eNOS and mechanosensor proteins in young and old men before and after training. Total protein expression, phosphorylation at serine^1177^ and relative phosphorylation of *endothelial nitric oxide synthase* (eNOS; panel **A–C**), and *platelet endothelial adhesion molecule 1* (PECAM-1, panel **D–F**) in whole muscle homogenates from young and old subjects at rest before and after the training intervention. #denotes difference from young group, ^∗^denotes difference from before training within group. Data are mean ± SD.

Before training, total eNOS protein amount and total eNOS^ser1177^ phosphorylation were similar in the two groups (Figure 2B) whereas the relative eNOS^ser1177^ phosphorylation was lower in the old than the young group (Figures 2A,C). After training, eNOS protein expression and total eNOS^ser1177^ phosphorylation were higher in the old and the young group (by 9 and 10%, and 4 and 15%, respectively; *P* < 0.05, Figure 2A). The relative eNOS^ser1177^ phosphorylation was not altered by training (Figure 2C).

#### VE-Cadherin and VEGF-R2

Before training, protein expression and relative phosphorylation levels of VE-Cadherin^tyr731^ and VEGF-R2^ser1175^ were not different between the young and old group and were not changed after the training period in either group (data not shown).

**Table 2:** Training data.

	Young	Old
Number of training sessions during the intervention	19 ± 3.6	20 ± 0.8
% of training time below 70% of HR_max_	31.7 ± 3.4	26.6 ± 6.8#
% of training time at 70–79% of HR_max_	25.1 ± 5.4	25.0 ± 7.2
% of training time at 80–89% of HR_max_	31.5 ± 3.9	36.2 ± 7.5
% of training time at 90–100% of HR_max_	11.6 ± 5.3	12.2 ± 5.4

*Data are presented as mean ± SD. HR, heart rate. #Significantly different from the young group (P < 0.05).*

#### PECAM-1

Before training, PECAM-1 protein levels were 23% higher in the old compared to the young group (*P* = 0.008, Figure 2D). After the training period, PECAM-1 protein expression was higher (by 43 and 85% respectively; *P* < 0.001) in both the young and old group and the level was 58% higher in the old compared to the young group (*P* = 0.013). Absolute PECAM-1^tyr713^ phosphorylation was not different between the young and old before or after training (Figure 2E) The relative phosphorylation of PECAM-1^tyr713^ was lower in the old compared to the young group before and after training (by 35 and 42% respectively, *P* < 0.001, Figure 2F) and was reduced by 29% in the old group after training (*P* = 0.04).

### Phosphorylation of eNOS and Mechanosensor Proteins With Passive Leg Movement

**FIGURE 3:**
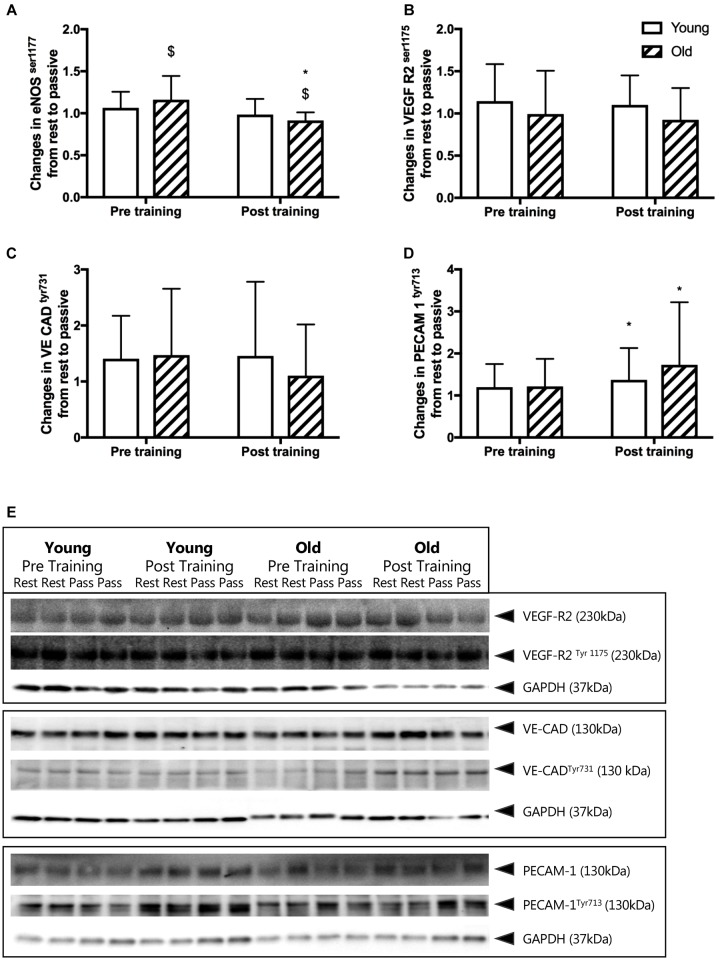
**(A–D)** Changes in relative phosphorylation of eNOS and mechanosensor proteins from rest to passive leg movement conducted before and after the training period. Relative changes in **(A)** *endothelial nitric oxide synthase serine^1177^* phosphorylation (eNOS ^p117^), **(B)** *vascular endothelial growth factor receptor 2 serine^1177^* (VEGF-R2^ser1177^), **(C)** *vascular endothelial cadherin tyrosine^731^* (VE CAD^tyr731^), and **(D)** *platelet endothelial adhesion molecule 1 tyrosine^713^* (PECAM-1^tyr713^) from young and old subjects before and after the training intervention. $ denotes a significant change from rest to passive leg movement, ^∗^denotes difference from before training within group. Data are mean ± SD. **(E)** Representative western blots of mechanosensor proteins. Western blots from one young and one old subject, before and after training of VEGF-R2^ser1177^, VE CAD^tyr731^ and PECAM-1^tyr713^. Samples are run in dublicats and presented together with loading control *glyceraldehyde 3-phosphate dehydrogenase* (GAPDH). Note that changes in eNOS phosphorylation are obtained from immunohistochemical staining of individual microvessels within cross sections of muscle biopsies while the mechanosensor proteins reflect changes in whole muscle homogenates. Please see the Materials and Methods section and Figure 5 for representative images.

Before training, passive leg movement increased the relative phosphorylation of eNOS^ser1177^ in the old by 16%, (*P* = 0.017, Figure 3A) but not in the young group. After training, passive leg movement led to a reduction of relative phosphorylation of eNOS^ser1177^ in the old group by 9% (*P* = 0.010) whereas it remained similar in the young group.

Before training, the relative phosphorylation of PECAM-1^tyr713^, VE-Cadherin^tyr731^ and VEGF-R2^ser1175^ was not changed by passive leg movement in either the young or the old group (Figures 3B–D). After training, passive leg movement increased phosphorylation of PECAM-1^tyr713^ in both the young and the old group by 37 and 73% (*P* = 0.011, Figure 3D).

**FIGURE 4:**
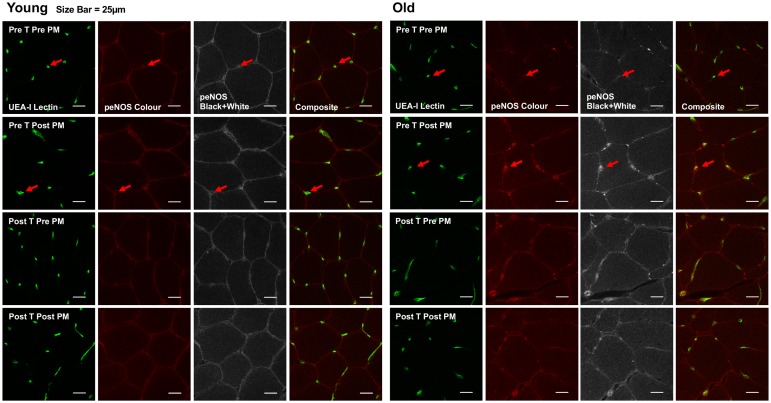
Representative images of immunofluorescence staining. Representative confocal microscopy images of skeletal muscle from one young and one old subject, before and after passive leg movement (Pre/Post PM) and before and after training (Pre/Post T). The skeletal muscle microvascular endothelium was revealed using *Ulex europaeus*-FITC conjugated lectin (UEA-I Lectin, green). Skeletal muscle eNOS^ser1177^ phosphorylation was revealed using Alexa Fluor 546 conjugated secondary antibody (peNOS Color, red). Scale bar represent 25 μm. Arrow indicates where positive staining for both endothelial specific Ulex and eNOS^ser1177^ phosphorylation is identified for quantification.

**FIGURE 5:**
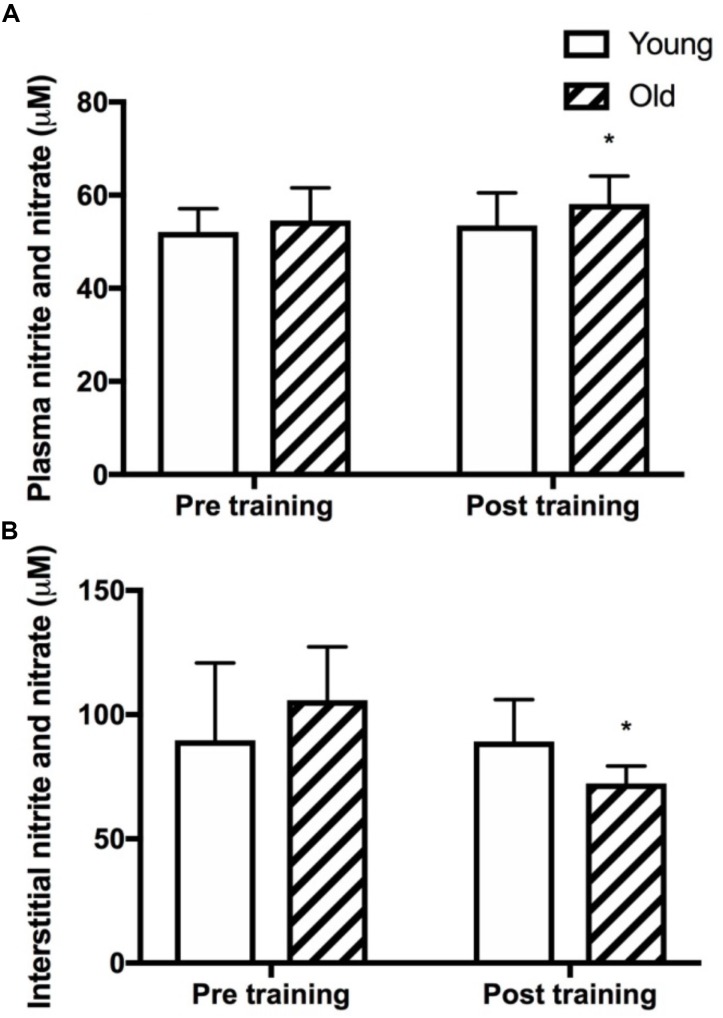
Plasma and skeletal muscle interstitial nitrite and nitrate levels. Plasma **(A)** and muscle interstitial **(B)** levels of the stable metabolites of nitric oxide, nitrite and nitrate (NOx) at rest in young and old subjects before and after the training intervention. ^∗^denotes different from before training within group. Data are mean ± SD.

Representative blots of the mechanosensors are presented in Figure 4 and quantitative immunofluorescence in Figure 5.

### Plasma and Interstitial NOx

Plasma NOx was not different between the young and the old group before training and was significantly increased only in the old group after training (*P* = 0.03, Figure 5A). Resting interstitial NOx was not different between the young and the old group before or after training but was lower after training in the old group (*P* = 0.05, Figure 5B).

## Discussion

This study is the first to examine the effect of aging and exercise training on expression and shear stress induced activation of endothelial mechanosensors. The principal findings are: (I) before training, compared to the young group, the older group presented a lower hyperaemic response to increased shear stress induced by passive leg movement, which was accompanied by a lower basal relative phosphorylation status of the endothelial mechanosensor PECAM-1; (II) eight weeks of exercise training resulted in a higher PECAM-1 protein level and an enhanced passive leg movement induced phosphorylation of PECAM-1 in both the young and the older subjects; (III) exercise training resulted in a higher expression of eNOS and a greater total eNOS phosphorylation in both young and aged individuals; (IV) after training, the hyperemic response to passive leg movement was higher in the young subjects. Combined, the results provide novel evidence for that the mechanosensor protein PECAM-1 is plastic in that both the protein amount and the phosphorylation status appear to be sensitive to aging and the level of physical activity. Although the changes in PECAM-1 observed with training were not paralleled by an improved hyperemic response during passive limb movement, there was a greater increase in phosphorylation of PECAM-1 for the same level of hyperemia in the old group, suggesting improved shear stress responsiveness.

### Vascular Function and the Mechanosensor Complex in Older Subjects

There is convincing evidence for that reduced availability of NO plays a central role in the age induced impairment in vascular function (Taddei et al., 2001) including the hyperaemic response to passive leg movement (Mortensen et al., 2012; Trinity et al., 2015). Nevertheless, the actual eNOS protein amount in skeletal muscle tissue has consistently been shown to be similar in old and young subjects (Donato et al., 2009; Nyberg et al., 2012a). This notion was also confirmed in the current study where both eNOS protein levels and the relative phosphorylation at rest were similar between the young and old group. Thus, either impaired eNOS activation upon stimulus, or removal of NO by reactive oxygen species, would be more plausible explanations for a reduced NO availability in older subjects. In this study we evaluated for the first time whether shear stress induced activation of the mechanosensor complex and eNOS is lower in old than in young individuals.

As functional model we utilized the hyperaemic response to passive leg movement, in which the flow response is shear stress induced and fully NO dependent (Mortensen et al., 2012). In accordance with previous observations, there was a lower blood flow response to passive movement in the old compared to the young subjects (Mortensen et al., 2012; Trinity et al., 2015). However, in contrast to our hypothesis, the activation of the mechanosensor complex with passive leg movement, as determined by a change in phosphorylation of the involved proteins, was not lower in the old compared to the young subjects. Moreover, there was no direct coupling between increased shear stress and eNOS activation, as eNOS^ser1177^ phosphorylation was increased in the old but not the young subjects with passive leg movement. Although our data support that eNOS can be phosphorylated by shear stress in older subjects, overall these findings only partially support *in vitro* studies showing activation of the mechanosensor complex and eNOS in response to shear stress (Fleming et al., 2005; Tzima et al., 2005; dela Paz et al., 2013). The reason for this discrepancy between *in vitro* and the present human findings remains unclear but could potentially be due to the many differences between *in vivo* and *in vitro models* such as the magnitude of shear stress. Nevertheless, an interesting novel observation in the present study was that the PECAM-1 protein expression was higher and the relative phosphorylation of PECAM-1 correspondingly lower in the old compared to the young subjects. These observations could indicate that vascular dysfunction is associated with an impairment in the basal state of PECAM-1 activation and it may be speculated that a compensatory upregulation of PECAM-1 occurs, similarly to that which appears to occur for eNOS (Donato et al., 2009; Nyberg et al., 2012a).

The basal protein expressions and degree of phosphorylation of VEGF-R2 and VE-Cadherin were similar in young and old subjects. The reason for the change in phosphorylation of PECAM-1 but no change in these other two proteins of the mechanosensor complex is unclear but suggests that PECAM-1 may be more sensitive to shear stress *in vivo* than the other proteins and potentially more important for endothelial function. It should be noted that the dynamics of phosphorylation of the mechanosensors in response to fluid shear stress *in vivo* is unknown and it is possible that, in our study, the assessed protein phosphorylation with elevated blood flow was returned to baseline by the time of the biopsy. However, blood flow during shear stress has been shown to be elevated for 90 min during passive leg movement (Høier et al., 2013) and studies on cultured endothelial cells exposed to fluid shear stress for periods of time from minutes to hours show that the phosphorylation status of various mechanosensors is maintained (Tzima et al., 2005; Russell-Puleri et al., 2017).

### Effects of Exercise Training on Vascular Function and the Mechanosensor Complex

Exercise training is generally thought to improve vascular function and cross-sectional data suggest that older active and well-trained subjects express well preserved vascular function compared to younger subjects (Nyberg et al., 2012a; Groot et al., 2016). Moreover, vascular sensitivity to shear stress, as determined by the hyperaemic response to passive leg movement, is proportional to the fitness level in middle-aged men (Lundberg Slingsby et al., 2018). In the present study, exercise training increased the hyperaemic response to passive leg movement in the young but not in the old subjects. This is the first study to evaluate the effect of exercise training on the flow response to passive leg movement in young individuals. Considering that the vasodilatory response to passive movement is highly NO dependent, (Mortensen et al., 2012) a potential explanation for the higher flow response in the young subjects after the training period could be a greater NO availability. Accordingly, the basal eNOS protein expression and the total eNOS^ser1177^ phosphorylation were increased in the young group after training. Thus, despite the fact that the change in eNOS phosphorylation with passive movement was similar before and after training, there was an enhanced potential of NO formation through the greater total amount of phosphorylated eNOS. This finding provides one explanation for the greater hyperemia after training in the young, however, although the aged group also showed increased eNOS expression and total eNOS^ser1177^ phosphorylation, they did not show a parallel improvement in shear stress induced hyperemia. A potential explanation for this observation could be that NO bioavailability is more dependent on removal of NO by reactive oxygen species in aged than in young (Nyberg et al., 2014) and that eNOS activity also is dependent on a number of other factors like other phosphorylations sites, co-factors and coupling status of the homodimeric enzyme (Fleming and Busse, 1999).

It should be noted that basal levels of the stable metabolites of NO, nitrite and nitrate (NOx) were assessed in plasma and interstitial fluid before and after the training period but there was no direct relationship to eNOS level and eNOS phosphorylation. This lack of relationship may be due to that NOx is a rather rough estimate of NO bioavailability and the levels are affected by diet which was not controlled for in the current study.

Out of the three proteins in the mechanosensor complex, the PECAM-1 protein content was enhanced in both the young and the old group after the training period. This increased PECAM-1 protein expression was paralleled by an increase in the passive leg movement induced relative PECAM-1^tyr713^ phosphorylation in both groups. PECAM-1 has several other additional physiological roles of this protein in the vasculature (Privratsky and Newman, 2014). PECAM-1 is known to be of relevance in physiological processes such as platelet function (Newman and Newman, 2003) angiogenesis and vascular repair processes (DeLisser et al., 1997; Biswas et al., 2003). Moreover, PECAM-1 in combination with VE-CAD and VEGFR2 regulate vascular integrity and permeability allowing for leukocyte extravasation (Hashimoto et al., 2011; Chistiakov et al., 2016). The present data suggest that physical activity is associated with improved expression of this molecule. Moreover, although microcirculatory shear stress cannot be assessed in humans *in vivo*, the fact that the hyperemic response to passive leg movement was similar in the old group after training compared to before, whereas the change in PECAM-1 phosphorylation was increased, suggest a similar degree of shear stress and thus an enhanced shear stress induced activation. Although we could not detect an association with eNOS phosphorylation, the evident sensitivity of PECAM-1 both to aging and training provides evidence in humans for that this protein may be an important molecule in the development of vascular disease as proposed based on numerous *in vitro* studies (Privratsky and Newman, 2014). The need for further studies on the role of PECAM-1 and other mechanosensors in humans is however, warranted. With regard to the two fold increase in PECAM-1 protein expression in the old group it is worth noticing the large standard deviation within the group. This observation of large individual variation in PECAM-1 protein amount was consistent in the two different biopsies take from each subject at the same timepoint. Moreover, 13 out of 13 old subjects and 11 out of 14 young subjects showed an increase in PECAM-1 protein expression and thus the effect of exercise training on this molecule appears very consistent.

### Study Limitation

Passive leg movement was used to induce shear stress without muscle metabolic signaling. However, passive leg movement only induced activation of PECAM-1 and not VE-CAD, or VEGF-R2 and it is possible that a stronger shear stress stimulus or added metabolic signaling by active exercise, would activate the mechanosensor complex to a greater extent. It is also plausible that other mechanosensors, e.g., luminal G protein receptors and integrins also plays a functional role in determining the hyperemic response to passive leg movement. The older subjects recruited to the current study were healthy as the purpose was to evaluate the effect of age *per se*. Accordingly, they had good cardiovascular health and it is plausible that greater differences in the mechanosensor complex and in the hyperaemic response to passive movement would be observed in older individuals with poorer health status.

It could be speculated that the level of capillarization explains the differences and changes in PECAM-1. Shear stress is dependent on blood flow, blood vessel diameter and viscosity of the blood. In using an *in vivo* model in humans the drawback is that the potential differences in the size and number of the arterioles in the microcirculation, as well as the perfusion pattern, in the muscle is unknown and thereby its potential influence on the actual shear stress. However, aging has been associated with a lower capillarization (Ryan et al., 2006) and, it is likely that the number of arterioles also may have been lower. This could potentially mean that the aged would have a stronger shear stress signal for a given level of flow. However, as all measured mechanosensors are endothelial cell specific and only PECAM-1 differed, this explanations seems unlikely.

## Conclusion

Our results demonstrate that the impaired shear stress induced hyperemia in aged is not immediately explained by a lower eNOS activation but there appears to be an association with the phosphorylation status of the important mechanosensor protein PECAM-1. Moreover, that the finding that exercise training leads to enhanced PECAM-1 phosphorylation in response to acute passive leg movement, despite a similar increase in blood flow, agrees with our hypothesis that the responsiveness of mechanosensory proteins can be improved by training. Overall, our study provides the first *in vivo* evidence in humans for plasticity of PECAM-1 and improvements in mechanosensing; factors which are likely to contribute to the known beneficial effects of physical activity on vascular function. The lack of association between the hyperemic response to passive leg movement and eNOS activation nevertheless warrants further investigation.

## Author Contributions

LG and YH conceived and designed the study. LG, NR, MC, and YH analyzed and interpreted the data. All authors collected the data, drafted the article or revised it critically for important intellectual content, approved the final version, and agreed to be accountable for all aspects of the work in ensuring that questions related to the accuracy or integrity of any part of the work are appropriately investigated and resolved.

## Conflict of Interest Statement

The authors declare that the research was conducted in the absence of any commercial or financial relationships that could be construed as a potential conflict of interest.

## Abbreviations

eNOS: endothelial nitric oxide synthase
NO: nitric oxide
PECAM-1: platelet endothelial cell adhesion molecule
ROS: reactive oxygen species
VEGF-R2: vascular endothelial growth factor receptor 2
VE-cadherin: vascular endothelial cadherin.
