# Supplementary figures and images for: The Endothelial Mechanotransduction Protein Platelet Endothelial Cell Adhesion Molecule-1 Is Influenced by Aging and Exercise Training in Human Skeletal Muscle
Source: Front Physiol. 2018 Dec 18;9:1807. doi: 10.3389/fphys.2018.01807 (PMC6305393; doi:10.3389/fphys.2018.01807)

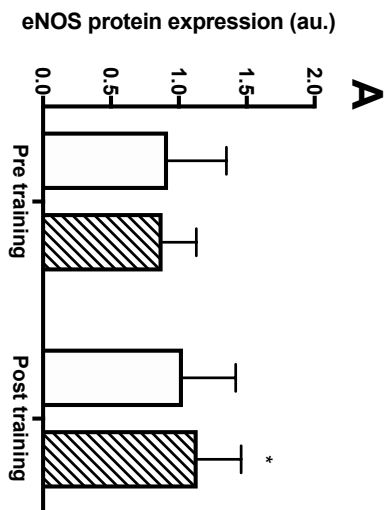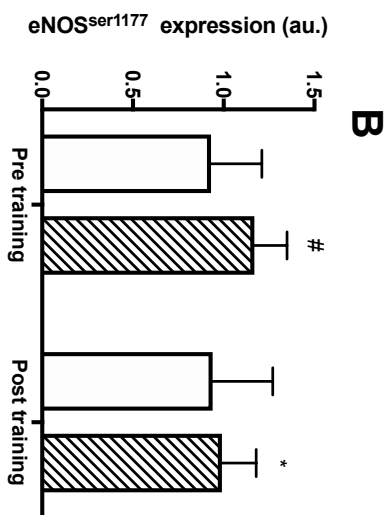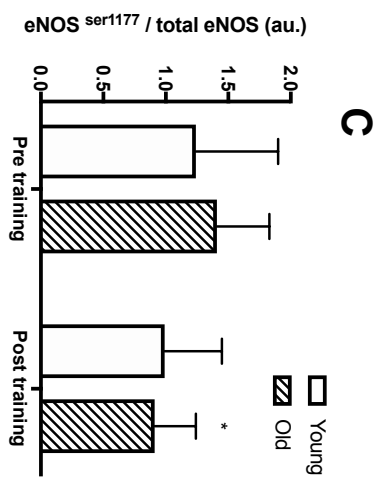

Supplement: Supplementary file 1 [file Image_1.pdf]

Absolute blood flow

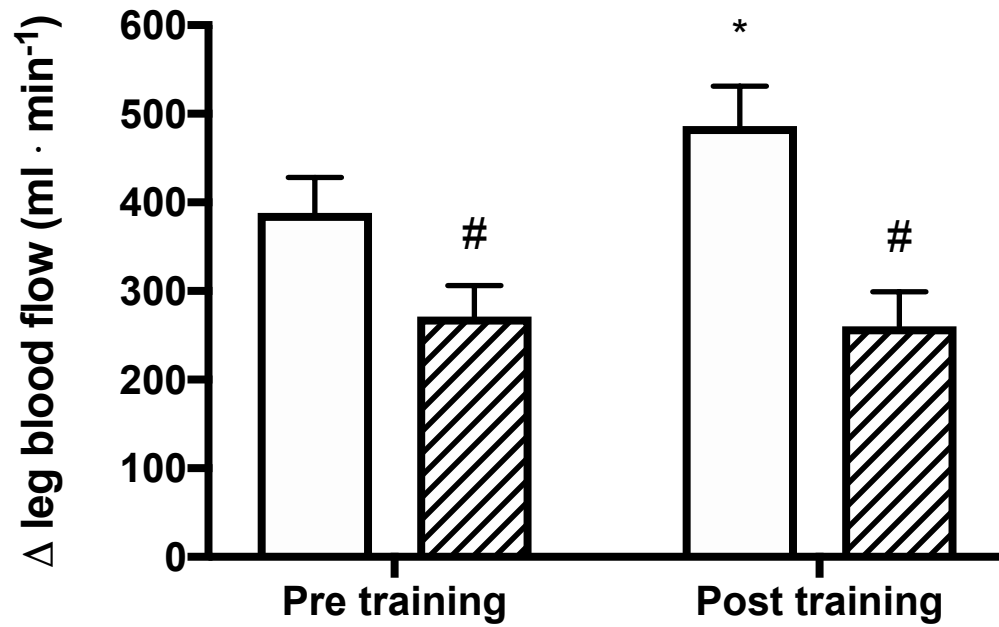

Blood flow normalized to leg mass

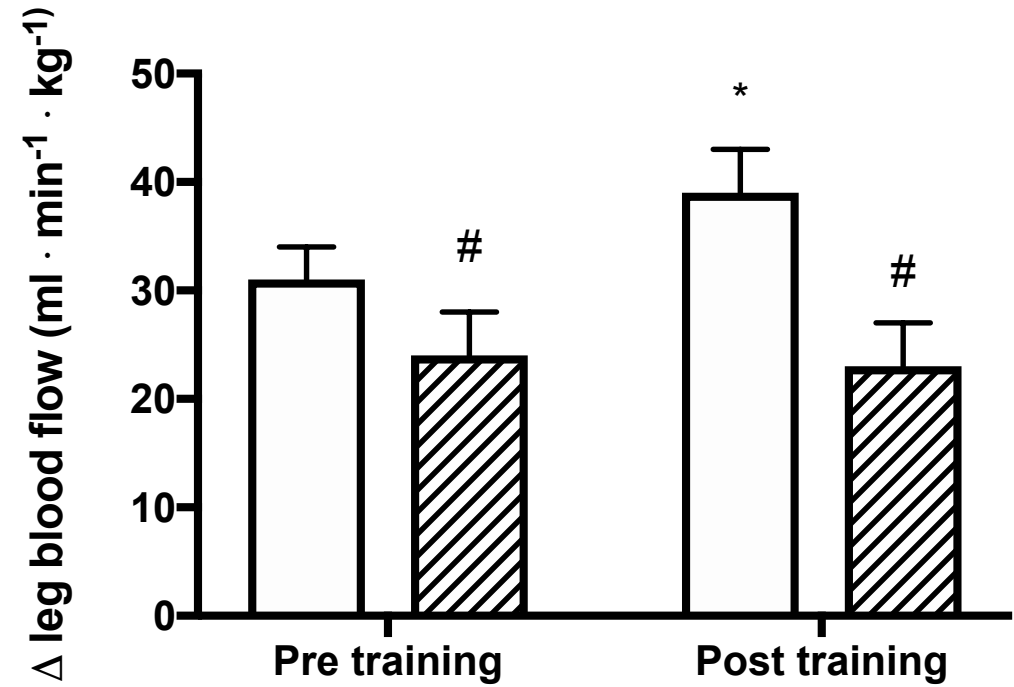

Supplement: Supplementary file 3 [file Image_3.pdf]
