# Supplementary material for: The Endothelial Mechanotransduction Protein Platelet Endothelial Cell Adhesion Molecule-1 Is Influenced by Aging and Exercise Training in Human Skeletal Muscle
Source: Front Physiol. 2018 Dec 18;9:1807. doi: 10.3389/fphys.2018.01807 (PMC6305393; doi:10.3389/fphys.2018.01807)

Figure S2
Stain free gel quantification

	Time	Condi	#	Adj vol.
YOUNG	Pre	Rest	***49***	97.179.211,56
	Pre	Pass	***50***	93.129.658,76
	Post	Rest	***51***	93.951.835,60
	Post	Pass	***52***	90.909.946,03
OLD	Pre	Rest	***53***	90.406.697,92
	Pre	Pass	***54***	91.122.484,00
	Post	Rest	***55***	80.988.768,25
	Post	Pass	***56***	88.400.635,44
Average across all samples within each group				
YOUNG				88.951.586,95
OLD				85.801.467,78

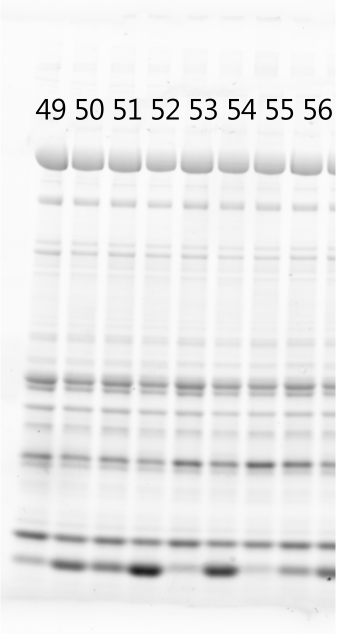

Supplement: Supplementary file 2 [file Image_2.pdf]
