# Supplementary material for: The Endothelial Mechanotransduction Protein Platelet Endothelial Cell Adhesion Molecule-1 Is Influenced by Aging and Exercise Training in Human Skeletal Muscle
Source: Front Physiol. 2018 Dec 18;9:1807. doi: 10.3389/fphys.2018.01807 (PMC6305393; doi:10.3389/fphys.2018.01807)
Supplement: Supplementary file 4 [file Data_Sheet_1.docx]

Supplemental figure legends

Figure s1. **Protein expression and relative phosphorylation of eNOS in young and old men before and after training.** Total protein expression, phosphorylation at serine 1177 and relative phosphorylation of *endothelial nitric oxide synthase* (eNOS; panel **A,** **B** and **C**), in whole muscle homogenates from young and old subjects at rest before and after the training intervention. # denotes difference from young group, * denotes difference from before training within group. Data are mean ± SD.

Figure s2. **Representative image of Stain free gel and total protein quantification.**

Figure s3. **Comparison of femoral arterial blood flow during passive leg movement normalized to leg mass. Left panel:** Changes in absolute femoral arterial blood flow from rest to passive leg movement. **Right panel:** Changes in femoral arterial blood flow relative to leg mass going from rest to passive leg movement. # denotes difference from young group, * denotes difference from before training within group. Data are mean ± SD.
